# Optimal Pore Size of Honeycomb Polylactic Acid Films for In Vitro Cartilage Formation by Synovial Mesenchymal Stem Cells

**DOI:** 10.1155/2021/9239728

**Published:** 2021-08-02

**Authors:** Misaki Yagi, Mitsuru Mizuno, Ryota Fujisawa, Hisako Katano, Kentaro Endo, Nobutake Ozeki, Yuriko Sakamaki, Hideyuki Koga, Ichiro Sekiya

**Affiliations:** ^1^Center for Stem Cell and Regenerative Medicine, Tokyo Medical and Dental University, 1-5-45, Bunkyo-ku, Yushima, Tokyo, Japan; ^2^Research Core, Tokyo Medical and Dental University, Tokyo, Japan; ^3^Department of Joint Surgery and Sports Medicine, Tokyo Medical and Dental University, Tokyo, Japan

## Abstract

**Background:**

Tissue engineering of cartilage requires the selection of an appropriate artificial scaffold. Polylactic acid (PLA) honeycomb films are expected to be highly biodegradable and cell adhesive due to their high porosity. The purpose of this study was to determine the optimal pore size of honeycomb PLA films for in vitro cartilage formation using synovial mesenchymal stem cells (MSCs).

**Methods:**

Suspensions of human synovial MSCs were plated on PLA films with different pore sizes (no pores, or with 5 *μ*m or 20 *μ*m pores) and then observed by scanning electron microscopy. The numbers of cells remaining in the film and passing through the film were quantified. One day after plating, the medium was switched to chondrogenic induction medium, and the films were time-lapse imaged and observed histologically.

**Results:**

The 5 *μ*m pore film showed MSCs with pseudopodia that extended between several pores, while the 20 *μ*m pore film showed MSC bodies submerged into the pores. The number of adhered MSCs was significantly lower for the film without pores, while the number of MSCs that passed through the film was significantly higher for the 20 *μ*m pore film. MSCs that were induced to form cartilage peeled off as a sheet from the poreless film after one day. MSCs formed thicker cartilage at two weeks when growing on the 5 *μ*m pore films than on the 20 *μ*m pore films.

**Conclusions:**

Honeycomb PLA films with 5 *μ*m pores were suitable for in vitro cartilage formation by synovial MSCs.

## 1. Background

Tissue engineering of cartilage requires the appropriate selection of cells [1]. Several cell candidates are currently available, including chondrocytes, induced pluripotent stem (iPS) cells, and mesenchymal stem cells (MSCs). The use of chondrocytes is invasive, as cell collection requires that normal cartilage be sacrificed [2]; however, iPS cells require more time and effort than other cell types for cartilage differentiation [3]. MSCs are therefore more useful, as their cell sources are easy to harvest, the cells proliferate well, and they can be induced to differentiate into cartilage. Synovial MSCs are particularly attractive as a cell source for tissue engineering of cartilage because of their high chondrogenic differentiation potential [4, 5].

The appropriate selection of artificial material is also important for cartilage engineering [6] in addition to the selection of synovial MSCs. Several artificial scaffold materials are already in clinical use [7]. In the field of orthopedics, one of the most popular scaffold materials is polylactic acid (PLA) because of its biodegradability; however, cells can have difficulty adhering to it. For this reason, PLA is not yet in common use as a scaffold for cells used clinically for cartilage regeneration. Efforts made to overcome this adhesion problem have included spinning of PLA nanofibers and arranging PLA fibers in lattice patterns [8, 9], but the types of PLA scaffolds that are most suitable for tissue formation have not yet been identified.

PLA can be prepared in the form of honeycomb-like sheets, and these are expected to show highly biodegradability and improved cell adhesion due to their high porosity [10]. Porous films can be formed from water droplet templates by the breath figure method, and this method has attracted considerable interest because of its simplicity and wide applicability to a variety of materials. Recently, honeycomb films have been prepared by the breath figure technique [11, 12]; however, the best pore size for efficient cell adhesion and cartilage formation has not been established. The purpose of the present study was to determine the optimal pore size of honeycomb PLA films for in vitro cartilage formation by synovial MSCs.

## 2. Methods

### 2.1. Preparation of Synovial MSCs

All methods were carried out in accordance with relevant guidelines and regulations. All procedures performed in the study involving human participants were in accordance with the Declaration of Helsinki [13]. This study was approved by the Medical Research Ethics Committee of Tokyo Medical and Dental University (M2017-142), and informed consent was obtained from all study subjects.

Human synovium was harvested from the knees of patients with osteoarthritis who underwent total knee arthroplasty operations, and cell culture was performed according to the method established in our previous reports [4, 14, 15]. Briefly, the synovium was minced and digested at 37°C for 3 h in a solution of 3 mg/mL collagenase (Sigma Aldrich, MO, USA), and the digested cells were filtered through a 70 *μ*m cell strainer (Greiner Bio-one GmbH, Kremsmuenster, Austria). The obtained nucleated cells were cultured in 150 cm^2^ culture dishes (Nalge Nunc International, Thermo Fisher Scientific, MA, USA) in 18 mL alpha minimum essential medium (*α*MEM, Thermo Fisher Scientific) containing 10% fetal bovine serum (FBS, Thermo Fisher Scientific). The cells were treated with 0.25% trypsin-EDTA (Thermo Fisher Scientific) at 37°C for 5 min, harvested, and cryopreserved as passage 0. For cell culture, the frozen cells were slowly thawed, plated, and incubated for 4 days as passage 1. These passage 1 cells were then replated at 50 cells/cm^2^, cultured for 14 days, and the resulting passage 2 cells were used for analyses.

### 2.2. Differentiation Assays

The differentiation potential of the MSCs was evaluated as in previous studies [15–17]. Chondrogenesis was examined by suspending 1.25 × 10^5^ synovial MSCs in 0.5 mL chondrogenic induction medium consisting of Dulbecco's modified Eagle's medium (DMEM, Thermo Fisher Scientific) containing 10 ng/mL transforming growth factor-*β*3 (TGF-*β*3, Miltenyi Biotec, Bergisch Gladbach, Germany), 500 ng/mL bone morphogenetic protein 2 (BMP-2, Medtronic, MN, USA), 40 *μ*g/mL proline (Sigma-Aldrich), 100 nM dexamethasone (Fujifilm Wako Pure Chemical Corporation, Osaka, Japan), 100 *μ*g/mL pyruvate (Sigma-Aldrich), 50 *μ*g/mL ascorbate-2-phosphate (Fujifilm Wako Pure Chemical Corporation), and 50 mg/mL 1% ITS Premix (BD: Becton, Dickinson and Company, NZ, USA). The cells were pelleted by centrifugation at 500 × g for 10 min and then cultured for 21 days. After centrifugation, the pellets were sectioned and stained with toluidine blue (Fujifilm Wako Pure Chemical Corporation) for morphological analysis. Adipogenesis was determined by suspending 100 synovial MSCs in a 60 cm^2^ dish and culturing in culture medium for 14 days to produce cell colonies. The adherent cells were cultured for a further 21 days in adipogenic induction medium consisting of *α*-MEM supplemented with 100 nM dexamethasone, 0.5 mM isobutylmethylxanthine (Sigma-Aldrich), and 50 mM indomethacin (Fujifilm Wako Pure Chemical Corporation). Adipocytes were stained with oil red O (Muto Pure Chemicals, Tokyo, Japan).

Calcification was studied by plating 100 synovial MSCs in a 60 cm^2^ dish and culturing for 14 days in culture medium to allow formation of cell colonies. The adherent cells were further cultured in a calcification induction medium consisting of *α*-MEM supplemented with 50 *μ*g/mL ascorbic acid 2-phosphate, 10 nM dexamethasone, and 10 mM *β*-glycerophosphate (Sigma-Aldrich). After 21 days, calcification was assessed by alizarin red staining (Merck Millipore, MA, USA).

### 2.3. PLA Honeycomb Films

Circular PLA films with a diameter of 6 mm and thickness of 5 *μ*m were prepared as three types: a PLA film without any pores (0 *μ*m), one with 5 *μ*m pores, and one with 20 *μ*m pores. Before cell plating, the films were immersed in 70% ethanol, washed with phosphate-buffered saline (PBS, Thermo Fisher Scientific) for hydrophilization, and incubated with FBS overnight at 4°C.

### 2.4. Scanning Electron Microscopy (SEM)

Synovial MSCs (1 × 10^4^ cells) were suspended in 500 *μ*L *α*MEM with 10% FBS and plated on PLA films in a 24-well plate. After 2 h, the films were fixed in 2.5% glutaraldehyde (TAAB Laboratories and Equipment Ltd., Berks, England) for 2 h and washed overnight in 0.1 M PBS at 4°C. Each film specimen was then postfixed with 1% osmium tetroxide (TAAB Laboratories and Equipment Ltd.) for 2 h at 4°C and dehydrated in graded ethanol solutions (Fujifilm Wako Pure Chemical Corporation). After exchanging with 3-methyl butyl acetate (Fujifilm Wako Pure Chemical Corporation) and critical point drying, the specimen was coated with platinum and the surface was observed by SEM (S-4500, Hitachi Ltd., Tokyo, Japan) [18].

### 2.5. Surface Markers

Synovial MSCs (5 × 10^5^ cells) at passage 3 in 500 *μ*L *α*MEM containing 10% FBS were dropped onto films in the wells of a 24-well plate. A 1 mL volume of *α*MEM containing 10% FBS was added, and the cells were incubated for 24 h at 37°C and 5% CO_2_. Adherent cells were then detached by treating with 0.25% trypsin-EDTA for 5 min. The cells were suspended in Hank's balanced salt solution (HBSS, Thermo Fisher Scientific) at a density of 5 × 10^5^ cells/mL and treated for 30 minutes on ice with antibodies for CD14, CD29 (integrin *β*1), CD31, CD44, CD45, CD49c (integrin *α*3), CD49f (integrin *α*6), CD51/CD61 (integrin *α*v*β*3), CD61, CD73, CD106, and CD146 (all from BD). The data were analyzed by FACS Verse (BD) and FlowJo software (Tree Star Inc., OR, USA). Cells positively stained with Ghost Dye Violet 510 (Tonbo Biosciences, CA, USA) were removed as dead cells. Isotype controls were prepared as negative controls (BD). The expressions of CD29, CD49c, CD49f, and CD51/CD61 by cells seeded on plastic dishes (just before seeding on the film) were examined in the same manner.

### 2.6. Quantitative Evaluation of Adherent Cells

Synovial MSCs were stained with DiI (Thermo Fisher Scientific) and 3 × 10^3^ synovial MSCs suspended in 500 *μ*L *α*MEM with 10% FBS were plated on the PLA films in a 24-well plate. After 24 h, the films and plates were fixed with 10% neutral formalin (Fujifilm Wako Pure Chemical Corporation). The MSCs remaining on the PLA films and the MSCs passing through the film to the bottom of the plates were observed, and the numbers of the cells were counted from the images obtained with a microscope analysis system (BZ-X810, KEYENCE, Osaka, Japan). Partial images of 4 × 4 area of film and a 10 × 10 area of bottom were taken at 4x magnification of the objective lens and automatically combined using software (BZ-X analyzer, KEYENCE). The regions with a fluorescence intensity above a fixed value within the film or on the bottom were defined as cells, and the number of regions was counted using imaging software (BZ-X analyzer). The numbers of nonadherent cells were calculated from the numbers of adherent cells on film and bottoms.

### 2.7. In Vitro Cartilage Formation of MSCs Plated on PLA Films

A 500 *μ*L volume of *α*MEM with 10% FBS containing 5 × 10^5^ synovial MSCs was dropped onto films in the wells of a 24-well plate. A further 1 mL *α*MEM with 10% FBS was added, and the cells were incubated for 24 h. The medium was then switched to chondrogenic induction medium (this was considered time 0). The medium was exchanged every 1–3days.

### 2.8. Time-Lapse Images

Synovial MSCs seeded on PLA films were observed with a microscope (EVOS XL Core Cell Imaging System, Thermo Fisher Scientific) at time 0, 1, 3, and 5 days. The film and cell sheet areas at each time point were defined manually using ImageJ software (National Institutes of Health, MD, USA), and the area ratio was calculated.

### 2.9. Histology

Synovial MSCs seeded on PLA films were washed with PBS, fixed in 4% paraformaldehyde (PFA, Fujifilm Wako Pure Chemical Corporation) at 4°C for 1 h, and embedded in 2% agarose gel (Fujifilm Wako Pure Chemical Corporation). The framework of the PLA film was then removed, and the cylindrical agarose gel holding the PLA film was embedded in paraffin, sliced, stained with safranin-o/fast green (Fujifilm Wako Pure Chemical Corporation), and observed with a microscope (BZ-X810).

The cartilage thickness was measured by drawing a single line along the long axis of the cartilage, determining the midpoint of both ends of the cartilage on that line, and then drawing seven perpendicular lines 500 *μ*m on both sides of that midpoint. The midpoints of both ends of the cartilage were determined on each vertical line, and the minimum width through these points was determined. Finally, an average thickness of the cartilage and the coefficient of variance for cartilage thickness at 7 points were calculated using ImageJ.

### 2.10. Statistical Analysis

The Shapiro-Wilk test was used to confirm the normality of the data (*P* > 0.05). The analysis between the two groups were calculated by a paired Student's *t* test. Cell counts and time courses were statistically analyzed by two-way analysis of variance (ANOVA) with Tukey's multiple comparisons test using GraphPad Prism 8 software (GraphPad Software, CA, USA). All statistical analysis methods are described in the figure legends. Two-tailed *P* values of <0.05 were considered statistically significant.

## 3. Results

### 3.1. SEM Images of Honeycomb PLA Films after Plating MSCs

**Figure 1:**
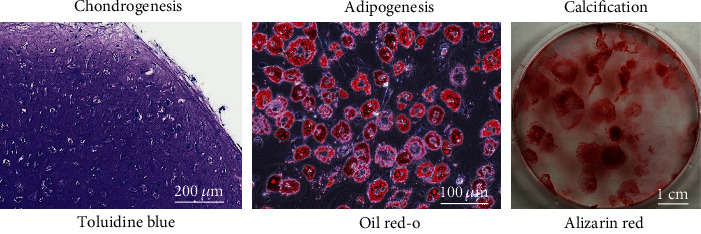
Multipotency of synovial MSCs. Synovial MSCs were cultured in chondrogenic, adipogenic, and calcification induction media. A section of the cartilage pellet was stained with toluidine blue, and the culture dishes for adipogenesis and calcification were stained with oil red O and alizarin red, respectively.

**Figure 2:**
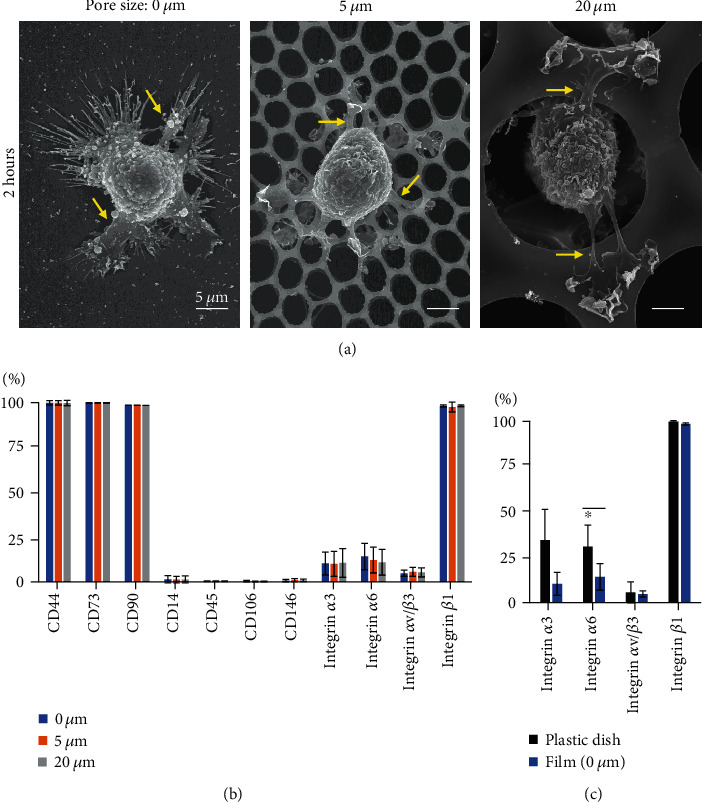
SEM images and surface markers of synovial MSCs plated onto honeycomb films. (a) SEM images of honeycomb PLA films after plating synovial MSCs. Synovial MSCs (1.0 × 10^4^ in 500 *μ*L medium) were plated on PLA films without pores (0 *μ*m) or with 5 *μ*m and 20 *μ*m pores and observed from above after 2 h. Representative pseudopodia are indicated with yellow arrows. (b) Surface markers of synovial MSCs one day after seeding onto the film. Means and standard deviations are shown (*n* = 3). (c) Surface markers of synovial MSCs before and one day after seeding onto 0 *μ*m film. ^∗^*P* < 0.05. *P* values were determined by paired Student's *t* test.

Synovial MSCs showed multipotency for chondrogenesis, adipogenesis, and calcification (Figure 1). At 2 h after plating onto the film without pores (0 *μ*m), MSCs showed extended pseudopodia and attachment to the film (Figure 2(a)). MSCs on the 5 *μ*m pore film also showed pseudopodia that extended and adhered to several pores. MSCs on the 20 *μ*m pore film showed submergence of the cell body into the pore and pseudopodia extending around the pore.

### 3.2. Surface Markers of Synovial MSCs Plated onto Honeycomb Films

One day after seeding onto films with pore sizes of 0, 5, and 20 *μ*m, the MSCs in each film expressed 100% of the positive MSC markers CD44, CD73, and CD90, and less than 5% of the negative MSC markers CD14, CD45, CD106, and CD146 (Figure 2(b)). The expression of the four different integrins in MSCs did not differ among the films, but the expression of integrin *α*6 was significantly lower in MSCs on films than in MSCs cultured in plastic dishes (Figure 2(c)).

### 3.3. MSC Numbers Remaining in and Passing through Films

**Figure 3:**
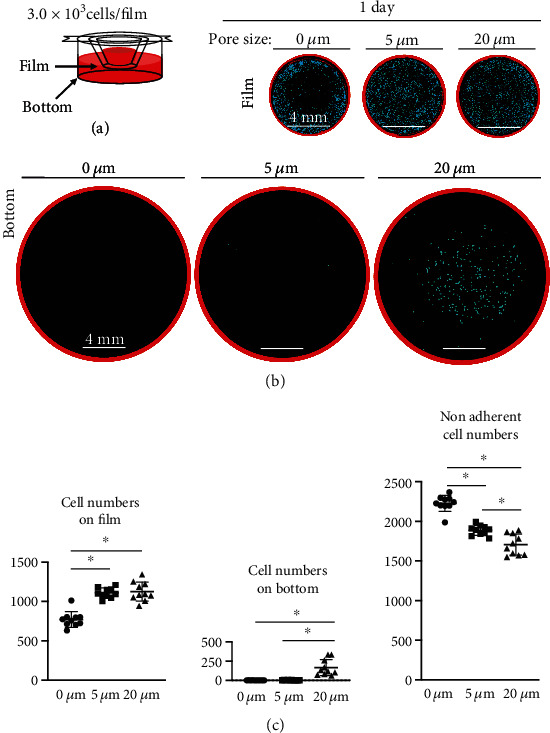
Analysis of numbers of synovial MSCs remaining in and passing through PLA films. (a) Experimental setting. (b) Images of MSCs remaining on PLA films and MSCs passing to the bottoms of the plates. DiI-stained cells were observed from above and image processed and are shown in blue. (c) Quantitative evaluation of cell numbers on the films, on the bottoms of the dishes, and nonadherent cells. Means and standard deviations are shown (*n* = 10). ^∗^*P* < 0.05. *P* values were determined by one-way ANOVA with Tukey's multiple comparisons test.

One day after seeding the MSCs (Figure 3(a)), fewer cells were observed in the film without pores than in the films with pores, and more cells were observed in the bottom of the dish containing the film with 20 *μ*m pores than dishes containing the other film types (Figure 3(b)). The number of cells on the film with no pores was 770 ± 100 cells, which was significantly lower than the cell number of 1110 ± 60 for the 5 *μ*m pore film or 1100 ± 120 cells for the 20 *μ*m pore film (Figure 3(c)). The number of cells on the bottom was essentially 0 cells for both the film with no pores and the 5 *μ*m pore film, which was significantly lower than 170 ± 110 cells noted for the 20 *μ*m pore film. The number of cells not adhered to the film or the bottom of the dish was 2200 ± 100 cells for the film with no pores and was significantly higher than the 1900 ± 60 cells obtained with the 5 *μ*m pore film. The value was also significantly higher than 1700 ± 130 cells obtained with the 20 *μ*m pore film.

### 3.4. The Early Phase of Cartilage Formation

**Figure 4:**
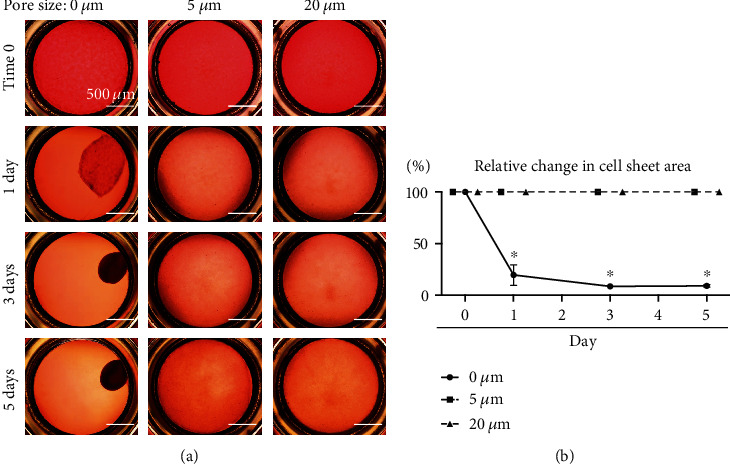
Early phase of cartilage formation by synovial MSCs on PLA films. (a) Time lapse pictures. MSCs (5.0 × 10^5^ in 500 *μ*L medium) were plated, cultured in chondrogenic induction medium, and then continuously observed. (b) Relative change in cell sheet area. Means and standard deviations are shown (*n* = 3). ^∗^*P* < 0.05. *P* values were determined by the two-way ANOVA with Tukey's multiple comparisons test.

During in vitro cartilage formation by MSCs, the cell sheet formed by the plated MSCs peeled off the film without pores at one day, assumed a round shape at three days, and was maintained as a cartilage mass at five days (Figure 4(a)). By contrast, the cell sheets formed by MSCs plated on the 5 and 20 *μ*m pore films remained sheet-like and did not peel off the film. Quantitative evaluation showed that the area of the sheet of MSCs plated on the film without pores significantly decreased to 20 ± 10% at 1 day, while the area of the sheet of MSCs plated on the film with 5 and 20 *μ*m pores was maintained at 100% for 5 days (Figure 4(b)).

### 3.5. Effect of Pore Size of the PLA Film on Cartilage Formation

**Figure 5:**
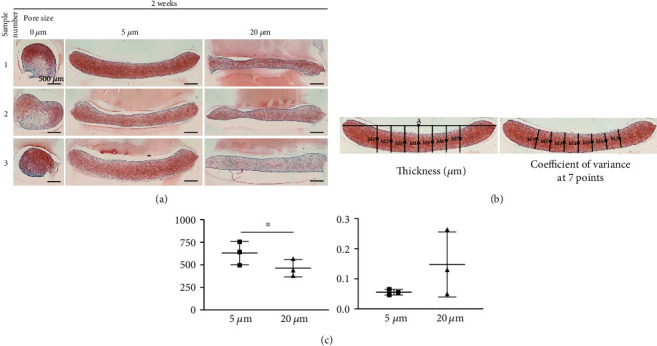
Histological images and thickness of cartilage derived from synovial MSCs plated on PLA films. (a) Histological images. MSCs (5.0 × 10^5^ in 500 *μ*L medium) were plated, cultured in chondrogenic induction medium for 14 days, and stained with safranin O. The results of three different samples are shown. (b) Method for measuring the cartilage thickness. A single line was drawn along the long axis of the cartilage, the midpoint of both ends of the cartilage was determined on this line (point A), and seven perpendicular lines were drawn every 500 *μ*m on both sides from “point A.” The midpoints of both ends of the cartilage were determined on each vertical line (M1-M7), and the minimum width through these points was determined. (c) Average thickness of the cartilage and the coefficient of variance for cartilage thickness at 7 points. Means and standard deviations are shown (*n* = 3). ^∗^*P* < 0.05. *P* values were determined by a paired Student's *t* test.

Synovial MSCs were plated on the three types of PLA films, cultured in the chondrogenic induction medium for two weeks, and then observed histologically (Figure 5(a)). MSCs plated on all three PLA films produced extracellular matrix, as confirmed by red staining with safranin O and they formed cartilage. The MSCs plated on the films without pores were spherical in shape, whereas the MSCs plated on the films with pores were sheet-like. The thickness of the sheet-like cartilage formed by MSCs plated on 5 *μ*m pore film (Figure 5(b)) was 630 ± 130 *μ*m and was significantly thicker than the 460 ± 100 *μ*m thick sheet-like cartilage formed by plating MSCs on the 20 *μ*m pore film (Figure 5(c)). The coefficient of variance for thickness of sheet-like cartilage measured at seven different points was 0.05 ± 0.01 for MSCs seeded on 5 *μ*m pore film and was significantly lower than the 0.15 ± 0.11 thickness measured for MSCs seeded on the 20 *μ*m pore film. This suggests the formation of a more even cartilage by MSCs seeded on 5 *μ*m pore film than on 20 *μ*m pore film.

### 3.6. Cartilage Formation of MSCs Plated on 5 *μ*m Pore PLA Films

**Figure 6:**
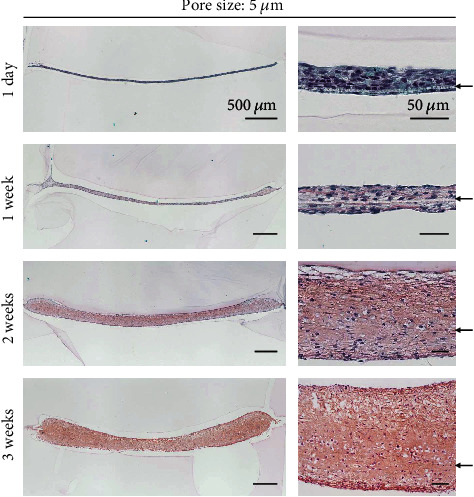
Temporal alteration in histological images of in vitro cartilage formation of synovial MSCs plated on PLA films with 5 *μ*m pores. MSCs (5.0 × 10^5^ in 500 *μ*L medium) were plated, cultured in chondrogenic induction medium for three weeks, and stained with safranin O. The arrow indicates the PLA film.

Synovial MSCs plated on 5 *μ*m pore PLA films and cultured in the chondrogenic induction medium for three weeks showed multilayered cells above the film and a small number of cells below the film (Figure 6). A few layers of cells were observed above and below the film and a slight cartilage matrix was produced at one week, and the thickness of the cartilage increased at two weeks and further increased to 300–500 *μ*m at 3 weeks. The thickness ratio of the cartilage above and below the film was approximately 3 : 1.

## 4. Discussion

Synovial MSCs seeded on PLA films without pores exhibited extended pseudopodia. Some reports have indicated that the pseudopodia perform the function of cell adhesion [18, 19]; however, in the present study, the number of adherent cells was on the film without pores was only about one third of the number on the PLA films with pores. This finding does not mean that the pseudopodia of synovial MSCs have no ability to adhere to films without pores; rather, it means that the pseudopodia of synovial MSCs are not sufficiently adhesive to withstand removal of the film from the dish, fixation of the cells with formalin, and microscopy observation. In other words, pores of a suitable size are useful to ensure efficient functioning of the pseudopodia.

The expression of surface markers by the synovial MSCs that adhered to the film did not differ with the presence or size of the pores, but the expression of integrin *α*6 was reduced compared to cells cultured on plastic dishes. The PLA film we used is a thin sheet of less than 10 *μ*m thickness and has softer physical properties than a plastic dish. The expression level of integrin *α*6 might be altered when MSCs adhere to soft PLA materials, since the expression levels of other integrins depend on mechanosensing of the rigidity of the materials by the adhering cells [20–24].

Chondrogenic induction was observed in MSCs plated on the PLA films without pores, but the cell sheet peeled off from the films after one day and formed a spherical cartilage mass after two weeks. The ascorbate-2-phosphate contained in the chondrogenic induction medium promotes the production of extracellular matrix and the aggregation of MSCs [25]. The observation of peeling of the cell sheets from the films during the process of cartilage formation indicates that the strength of aggregation of the MSCs is greater than the strength of adhesion of the MSCs to the PLA films without pores.

MSCs plated on 5 *μ*m pore films formed the thickest cartilage among the three types of films used in this study. This is because the MSCs did not fall through the pores after plating; instead, they firmly adhered to the pores via their pseudopodia. This firm adhesion prevented the cells from peeling off after chondrogenic differentiation. The chondrogenic differentiation of MSCs using scaffolds is affected by the size of the scaffold pores [26, 27]. In the current study, the film with the 5 *μ*m pore size was the best of the three tested films for cartilage formation by synovial MSCs under the conditions used here. MSCs seeded on 20 *μ*m pore films formed thinner and more uneven cartilage compared to MSCs seeded on 5 *μ*m pore films. The number of cells that adhered to the film one day after seeding was the same, but the number of cells that passed through the film and fell to the bottom was higher for the 20 *μ*m pore films.

Although the MSCs extended their pseudopodia and were caught in the pores, some of the cells passed through the 20 *μ*m pores, probably because the MSC body is less than 20 *μ*m in diameter [28] and therefore smaller than the pores. One possibility why the thickness and smoothness of the cartilage formed at 2 weeks differed between the films with 5 *μ*m and 20 *μ*m pores, even though the number of cells remaining in the film at 1 day was the same, might be that cells fell through the 20 *μ*m pores from 1 day to 2 weeks after seeding, especially in the early period before the cartilage matrix was fully formed.

The pore size in honeycomb films can be adjusted in a range from several tens of nanometers to tens of micrometers by controlling the template water droplets by the breath figure method. The honeycomb films have the added advantage of forming uniformly sized pores when compared to conventional porous scaffolds with size-distributed pores [11]. The pore sizes reported for honeycomb films have been found suitable for proliferation and function of endothelial cells [29], myocytes [30], and hepatocytes [31]. Kawano et al. compared PLA honeycomb films with 1.6 *μ*m, 3.2 *μ*m, and 4.7 *μ*m pores and reported that the number of adherent bone marrow MSCs increased as the pore size increased [32]. This finding concurred with our results and suggests that a suitable pore size for MSC adhesion to a PLA honeycomb film is approximately 5 *μ*m.

One limitation of our study is that we did not conduct in vivo investigations of the effectiveness of implanting honeycomb PLA films plated with MSCs into cartilage defects. Therefore, we cannot say at this time whether cartilage defects will be repaired when transplanted with undifferentiated MSCs adhering to 5 *μ*m pore honeycomb PLA films or whether the MSCs will require further ex vivo differentiation into cartilage sheets. We also do not know whether the PLA film will be absorbed after implantation, or when this will occur. Another point that requires clarification is whether a PLA film will be recognized as a foreign substance in the joint and cause inflammation.

We compared in vitro cartilage sheet formation by synovial MSCs using honeycomb PLA films with 0, 5, and 20 *μ*m pores. MSCs on the 5 *μ*m pore film showed pseudopodia that extended out to several pores. MSCs on the 20 *μ*m pore film showed cell bodies submerged in the pores. MSCs plated on 5 *μ*m pore films formed the thickest and most even cartilage layer among the three types of films. Honeycomb PLA films with 5 *μ*m pores were therefore considered suitable for in vitro cartilage formation by synovial MSCs.

## Data Availability

The datasets used and/or analyzed during the current study are available from the corresponding author on reasonable request.

## Abbreviations

PLA: Polylactic acid
MSCs: Mesenchymal stem cells.
